# Letter to the Editor: Enhancing Interpretation of AI Readiness in Medical Education

**DOI:** 10.1002/hsr2.71397

**Published:** 2025-10-16

**Authors:** Alvin Yong Zong Tung, Ley Wen Dong

**Affiliations:** ^1^ Mersey and West Lancashire Teaching Hospitals Foundation Trust Prescot UK

## Transparency Statement

Mr Alvin Tung Yong Zong affirms that this manuscript is an honest, accurate, and transparent account of the study being reported; that no important aspects of the study have been omitted; and that any discrepancies from the study as planned (and, if relevant, registered) have been explained.

To the Editor,

We are writing in response to the article “Insights Into the Future: Assessing Medical Students' Artificial Intelligence Readiness—A Cross‐Sectional Study at Kerman University of Medical Sciences (2022)” by Rezazadeh, Mahani, and Salajegheh [1]. The authors provided valuable insights into medical students' readiness for AI integration into healthcare, particularly in the context of a developing country such as Iran. Additionally, we would like to commend the authors for providing a comprehensive summary comparison of AI readiness in medical students between various countries. In this letter, we wish to offer a few points for consideration regarding its methodology and interpretation, which could further strengthen future research in this critical area.

While the Medical Artificial Intelligence Readiness Scale for Medical Students (MAIRS‐MS) [2] is a validated and valuable tool in assessing AI readiness in medical students, the self‐reporting nature of the questionnaire results in potential overestimation of true competence. This is evidenced by a study in Saudi Arabia by Bin Dahmash et al., where, although 50% of respondents felt that they had a good understanding of AI, objective tests revealed an average of only one correct answer out of five [3]. This demonstrates a significant disparity between self‐reported students' confidence in their AI knowledge and their actual understanding. While the authors acknowledge this as a limitation, the absence of an objective assessment means that the reported “readiness” scores might not fully reflect actual competency. We suggest that future studies in this field incorporate a mixed‐methods approach that includes practical assessments or observational data to complement the MAIRS‐MS questionnaire.

Furthermore, the absence of a defined readiness cutoff limits the interpretability of the MAIRS‐MS results. This is particularly relevant given the authors' conclusion that Iranian medical students have low AI readiness, which was largely based on mean scores falling below the midpoint of the 5‐point Likert scale. We believe this conclusion may be an overgeneralization, especially considering that the students scored a relatively high 10.94 out of 15 (72.9%) in the ethics domain. Without an established threshold to differentiate between low, moderate, and high readiness, such a conclusion may be premature and should be interpreted with caution. At present, the primary utility of the MAIRS‐MS scores lies in comparison between different cohorts of medical students as well as tracking changes in readiness over time. In the future, establishing a cutoff score for the MAIRS‐MS scale would provide a standardized benchmark, enabling educators to make more informed decisions about curriculum development and targeted interventions.

Despite these considerations, we would like to commend the authors for their insightful study and contribution to the growing discourse on AI learning in medical education. Through the integration of objective assessments and defining clear benchmarks, future studies can build upon this foundation and more robustly assess AI readiness in medical students.

## Author Contributions

**Alvin Yong Zong Tung:** conceptualization, writing – original draft, writing – review and editing, methodology. **Ley Wen Dong:** conceptualization, writing – original draft, writing – review and editing; methodology.

## Disclosure

All authors have read and approved the final version of the manuscript. Mr Alvin Tung Yong Zong had full access to all of the data in this study and takes complete responsibility for the integrity of the data and the accuracy of the data analysis.

## Conflicts of Interest

The authors declare no conflicts of interest.

## Data Availability

Data sharing is not applicable to this article as no datasets were generated or analyzed during the current study.
